# Effect of lactulose intervention on gut microbiota and short chain fatty acid composition of C57BL/6J mice

**DOI:** 10.1002/mbo3.612

**Published:** 2018-03-24

**Authors:** Shixiang Zhai, Limeng Zhu, Song Qin, Lili Li

**Affiliations:** ^1^ Yantai Institute of Coastal Zone Research Chinese Academy of Sciences Yantai Shandong China; ^2^ University of Chinese Academy of Sciences Beijing China; ^3^ Institute of Process Engineering Chinese Academy of Science Beijing China

**Keywords:** 16S rRNA high‐throughput sequencing, gut microbiota, prebiotic, probiotics, short chain fatty acids

## Abstract

Gut microbiota have strong connections with health. Lactulose has been shown to regulate gut microbiota and benefit host health. In this study, the effect of short‐term (3 week) intervention of lactulose on gut microbiota was investigated. Gut microbiota were detected from mouse feces by 16S rRNA high‐throughput sequencing, and short chain fatty acids (SCFAs) were detected by gas chromatography‐mass spectrometry (GC‐MS). Lactulose intervention enhanced the α‐diversity of the gut microbiota; increased the abundance of hydrogen‐producing bacteria Prevotellaceae and Rikenellaceae, probiotics Bifidobacteriaceae and Lactobacillaceae, and mucin‐degrading bacteria *Akkermansia* and *Helicobacter*; decreased the abundance of harmful bacteria Desulfovibrionaceae and branched‐chain SCFAs (BCFAs). These results suggest that lactulose intervention effectively increased the diversity and improved the structure of the intestinal microbiota, which may be beneficial for host health.

## 1 INTRODUCTION

Human gut is inhabited by a mixture of bacteria, archaea, and fungi; there are about 10^14^ microorganisms in the gut, encoding more than 3 million genotypes. These microbiota represent 10 times in the number of human cells, and 100 times the genotype (Tidjani Alou et al., 2016, Vassallo et al., 2015). Gut microbiota can be divided into three major groups, namely beneficial bacteria (probiotics), neutral bacteria, and pathogens, and the balance of those microbiota plays a significant role in host health. Gut microbiota have coevolved with humans to participate in metabolism, nutrition, and immune and other physiological functions, which has played a very important role in the development of mankind (Martin et al., 2007).

Because of their great number and variety of function, gut microbiota may be thought of as a huge organ, and the human body as a symbiont consisting of microbiota and human cells (Lederberg, 2000). Gut microbial dysbiosis is the leading cause of numerous chronic (Murphy et al., 2015) and metabolic diseases (Cani and Delzenne, 2009). The composition of the gut microbiota can be influenced by many factors, such as lifestyle, region, age, gender, and diet (Sommer & Backhed, 2013; Yatsunenko et al., 2012).

Lactulose is a disaccharide isomerized from lactose (Aider & Gimenezvidal, 2012), which is widely available and cheap. Consumption of lactulose has been associated with a number of health benefits, including treatment of constipation, hepatic encephalopathy and tumor, and maintenance of blood glucose and insulin levels (Panesar & Kumari, 2011). As a type of prebiotic, lactulose is not broken down by mammalian enzymes in the intestine, but is metabolized by gut microbiota to short chain fatty acids (SCFAs) in the ileum (Guerra‐Ordaz et al., 2014). Lactulose can change the composition of the gut microbiota. For example, Vanhoutte et al. (2006), reported a significant increase in *Bifidobacterium adolescentis* following lactulose intake. Tuohy et al. (2002) showed that *Bifidobacterium* spp. were increased, whereas *Clostridia* and *Lactobacilli* were decreased after lactulose treatment in humans.

SCFAs are main metabolites of gut microbiota, and are divided into straight‐chain SCFAs and branched‐chain SCFAs (BCFAs). Straight‐chain SCFAs are mainly produced by microbial fermentation of unabsorbed dietary carbohydrates in the gut. Lactate and succinate can also be metabolized to straight‐chain SCFAs, including acetate, propionate, and butyrate (Hasebe et al., 2016; Verbeke et al., 2015). Straight‐chain SCFAs have a range of beneficial effects, including regulation of the colonic and intracellular environment (Wong et al. 2006), and modulation of cell proliferation and gene expression. In addition, straight‐chain SCFAs are able to improve immune function, glucose regulation, and prevent obesity (Polyviou et al., 2016). In contrast, BCFAs are always derived from catabolism of branched‐chain amino acids (Zheng et al., 2013), and are major markers of protein fermentation, which is likely to be detrimental to the host (Yang & Rose, 2015).

Although some studies have assessed the effects of lactulose on gut microbiota, the gel‐ or PCR‐based methods used limit our ability to evaluate the full extent of the impact of lactulose on the gut microbiotic community. In this study, 16S rRNA high‐throughput sequencing and gas chromatography‐mass spectrometry (GC‐MS) were used to evaluate effect of lactulose on gut microbiota and their metabolites in mice.

## 2 MATERIALS AND METHODS

### 2.1 Animals and experiment design

Six‐week‐old male C57BL/6J mice were purchased from Pengyue Laboratory Animal Company (Jinan, China). All mice were raised in a temperature and humidity‐controlled animal laboratory with food and water provided ad libitum throughout the whole study. Composition of the basic diet is shown in Table 1. After 7 days acclimatization, 16 mice were randomly separated into two groups based on body weight: the control group (CG, *n* = 6) and experimental group (EG, *n* = 10). In this study, EG mice were given a gavage of lactulose at dosage of 2.5 g·kg ^−1^·day^−1^. CG mice were given a gavage of distilled water, with the same volume as in the treatment of EG mice, once per day. At the start of the experiment (0 weeks) and after 3 weeks of lactulose intervention, mice were transferred individually to separate sterilized cages and feces were collected. This study was approved by the Animal Care and Use Committee of Binzhou Medical University (BMU No. 20 100 701‐1).

**Table 1:** Nutrient content of basal diet (g/kg basal diet)

Ingredient	Mass
Water	≤100
Crude protein	≥180
Crude fat	≥40
Crude fiber	≤50
Coarse ash	≤80
Calcium	10–18
Total phosphorus	6–12
Calcium: total phosphorus	1.2:1–1.7:1
Lysine	≥8.2
Methionine + Cystine	≥5.3

### 2.2 Determination of SCFAs in feces

Feces were collected from individual mice. Fecal samples (50 mg) were added to 2 ml water, acidified with sulfuric acid (10%) to adjust the pH to 2–3, after that shocked and resuspended for 2 min. Then, 1 ml diethyl ether was added; 10 min later, the sample was centrifuged at 1,800 g for 10 min to remove the solid material. Supernatants were retained, cyclohexanone solution in ether (0.1 ml of 1000 mg/L) was added as internal standard, and the solution was filtered by through a 0.45 μm microporous membrane. Samples were analyzed by GC‐MS within 24 h. 1 μL of sample was injected into GC‐MS, which was equipped with a DB‐Wax column. Helium was the carrier gas at a flow rate of 0.8 mL/min. The injection temperature was 180°C and the GC temperature program was as follows: begin at 140°C, increase to 160°C at 5°C/min, then hold at 160°C for 6 min. The ion source temperature was 200°C. Concentration of SCFAs, including BCFAs, were analyzed using Single Ion Monitor (SIM) scan mode, calculated using the internal standard method and expressed in g/kg sample.

### 2.3 DNA extraction and 16S rRNA gene sequencing

Fresh fecal samples were collected individually, and all the samples were frozen in liquid nitrogen and stored at −80°C until analysis. Fecal samples were thawed on ice and DNA was extracted using a QIAamp DNA Stool Mini Kit (QIAGEN) according to the manufacturer's instructions.

Then, the V3‐V4 region of bacterial 16S rRNA genes was amplified. Amplicons were sequenced on the Illumina MiSeq platform (Illumina Inc., San Diego, CA). Illumina paired‐end library preparation, cluster generation, and 2 × 300 bp paired‐end sequencing were performed in one runs. The following cut‐off values were used for taxonomic assignment: species (X ≥ 97%), genus (97% >X ≥ 94%), family (94% >X ≥ 90%), order (90% > X ≥ 85%), class (85% > X ≥ 80%), and phylum (80% > X ≥ 75%), where X corresponds to the sequence identity between sequences within operational taxonomic units (OTUs) (Chae, Pajarillo, Oh, Kim, & Kang, 2016).

### 2.4 Statistical analysis

The Mann–Whitney *U*‐test was used to identify differences between two groups. The analyses were performed using SPSS (version 19.0, SPSS Inc., Chicago). Significance was accepted with a *p* < .05.

## 3 RESULTS

### 3.1 DNA sequence data and quality control

**Figure 1:**
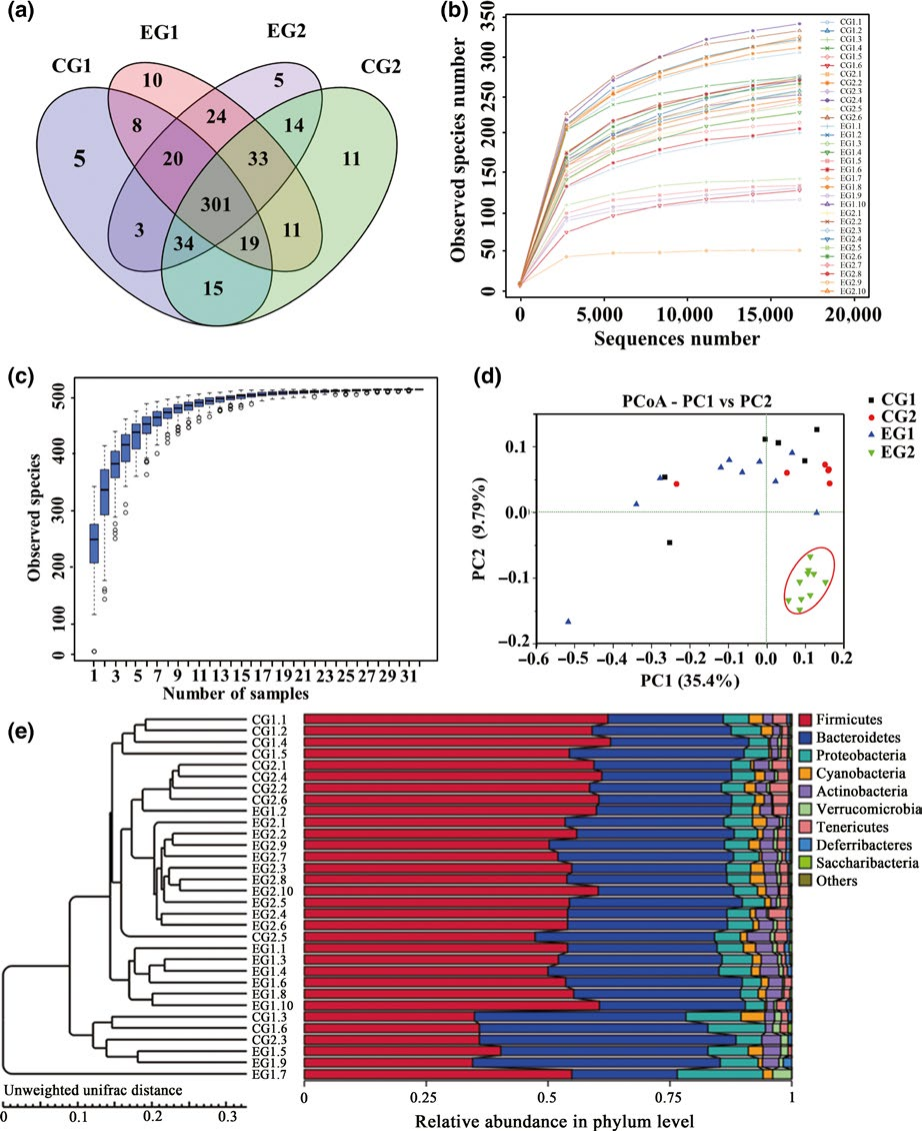
DNA sequences data and OTUs‐based community compositions in fecal microbiota before and after lactulose intervention. (a) Venn diagram of the OTUs for the CG, EG. Numbers indicated the number of OTUs that were unique and the number shared (core) by two or more groups, as depicted by no‐intersecting and intersecting ellipses, respectively. (b) Rarefaction analysis of the 32 different communities. (c) Species accumulation curves of the 32 different communities. (d) Variations of microbiota in CG and EG by PCoA analysis. (e) Unweighted‐pair group method with arithmetic mean tree of all subjects. CG, control group, *n* = 10; CG1, control group, week 0; CG2 control group, 3rd week; EG, experimental group, *n* = 6; EG1, experimental group, week 0; EG2, experimental group, 3rd week

A total of 1,129,104 sequences were obtained after pyrosequencing, and the average length was 35,284. Using 97% identity as the cutoff, 513 OTUs were delineated. In total, 405, 438, 426, and 434 OTUs were, respectively, obtained in CG1 (control group, week 0), CG2 (control group, 3rd week), EG1 (experimental group, week 0), and EG2 (experimental group, 3rd week). A Venn diagram was used to exhibit the different and common OTUs between groups (Figure 1a). The number of OTUs shared by at least two groups was 301. The rarefaction curves (Figure 1b) and species accumulation curves (Figure 1c) for all mice reached a plateau, indicating that the bacterial diversity in these communities was mostly covered.

Principal coordinate analysis (PCoA) of UniFrac distances based on the relative abundance of OTUs revealed that the microbiota shifted over time in each group (Figure 1d). The first two dimensions of the PCoA plots depicted unweighted UniFrac distances between microbial communities. The first (PC1) and second (PC2) axes contributed 35.4% and 9.79% of the variation, respectively. Each point represented the microbial community in a fecal sample from one mouse and community clustering illustrated an effect of lactulose. A PCoA score plot based on unweighted‐UniFrac distance showed that the 10 samples of EG2 were well separatedy from those of the other three groups, whereas almost all samples in CG1, CG2 and EG1 were distributed in the same area. This phenomenon showed that the overall microbiota was modulated in EG2 compared with the other three groups, whereas there was no significant difference between CG1 and CG2. This was further confirmed by an unweighted pair group method with arithmetic mean tree (Figure 1e).

### 3.2 α‐Diversity

3.2

Differences in gut microbial communities before and after lactulose intervention were measured by α‐diversity, which consists of richness estimates and diversity values. Richness estimates included Chao1 and the abundance‐based coverage estimate index, and diversity values included Shannon and Simpson indices. Qualified sequences reads were used to evaluate the diversity indices, in which higher quantities corresponded to higher diversity.

**Figure 2:**
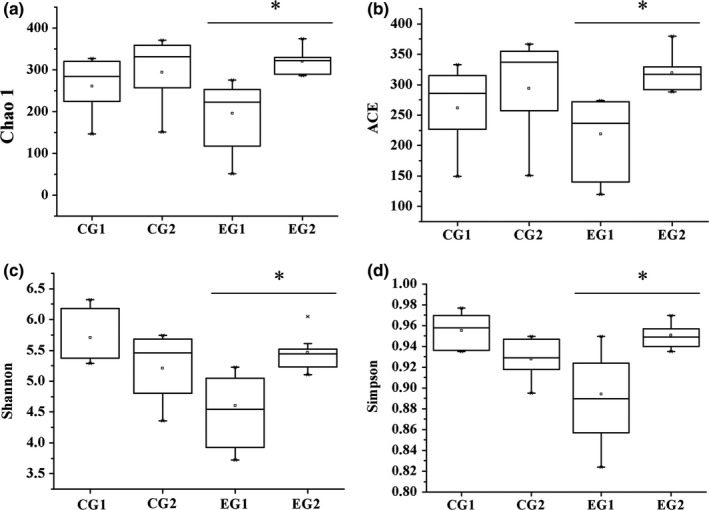
α‐diversity of C57BL/6J mice fecal microbiota after lactulose intervention for 3 weeks. Microbial richness estimates (ACE and Chao1) and diversity indices (Simpson and Shannon) were measured at OTUs definition of >97% identity. CG1, control group, week 0; CG2 control group, 3rd week, n = 10; EG1, experimental group, week 0; EG2, experimental group, 3rd week, n = 6. Data were analyzed by nonparametric test followed by Mann–Whitney *U*‐test. *p <* *.05

EG2 showed significantly higher diversity values and richness estimates than EG1 (Figure 2). Richness estimates increased and diversity values decreased in CG2 compared with CG1, but the differences were not statistically significant. Diversity analysis both in previous work in swine (Chae, Pajarillo, Park, & Kang, 2015) and in this study suggested that lactulose intervention could improve richness and diversity of gut microbiota.

### 3.3 OTUs analysis

**Figure 3:**
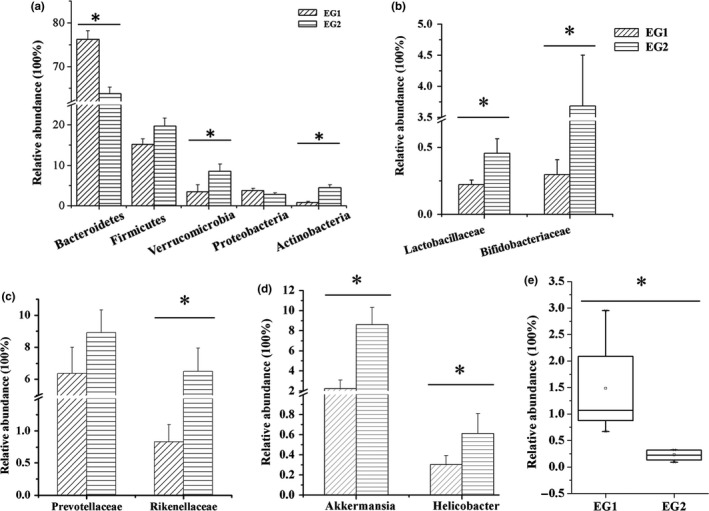
Fecal microbiota of mice before (left) and after (right) lactulose intervention. (a) The mean relative abundances of bacterial phyla in fecal samples before and after lactulose intervention. (b) The mean relative abundances of Lactobacillaceae and Bifidobacteriaceae before and after lactulose intervention. (c) The mean relative abundances of hydrogen‐producing bacterium before and after lactulose intervention. (d) The mean relative abundances of mucin‐degrading bacterium before and after lactulose intervention. (e) The mean relative abundances of Desulfovibrionaceae before and after lactulose intervention. EG1, experimental group, week 0; EG2, experimental group, 3rd week, *n* = 6. Data were analyzed by nonparametric test followed by Mann–Whitney *U*‐test. * *p *<* *.05

At phylum level, the most abundant sequences were members of the Bacteroides, Firmicutes, Verrucomicrobia, Proteobacteria, and Actinobacteria, dominated by Firmicutes and Bacteroides (>80%), both in EG1 and EG2 (Figure 3a). The abundance of Bacteroides decreased significantly after lactulose intervention (i.e., in group EG2), whereas the abundance of Firmicutes increased, therefore, the ratio of Firmicutes to Bacteroidetes increased after lactulose intervention. Meanwhile, the abundance of phyla Verrucomicrobia and Actinobacteria dramatically increased after lactulose intervention. These data indicated that lactulose treatment significantly influenced gut microbiota, and some phylum in particular.

At family level, a total of 38 bacterial families were detected in this study. Major microbiota groups detected in EG2 were: Bacteroidales_S24‐7_group (48.07%), Verrucomicrobiaceae (7.43%), Lachnospiraceae (9.02%), Erysipelotrichaceae (5.98%), Prevotellaceae (5.81%), and Rikenellaceae (5.81%) in EG2. After lactulose intervention (i.e., in EG2), the average populations of Lactobacillaceae, Bifidobacteriaceae, Prevotellaceae, and Rikenellaceae increased compared with EG1 (Figure 3b–c), particularly the Bifidobacteriaceae, which increased more than 10‐fold (from 0.3% to 3.7%). Desulfovibrionaceae decreased in EG during the experimental period (Figure 3e).

At the genus level, 87 bacterial genera were identified in this study. 33 differentially abundant genera (based on a cut‐off of >0.1% of total sequences) were detected, among which 12 genera each represented *x* > 1.0% of the genera sampled. The dietary lactulose increased the levels of mucin‐degrading bacteria *Akkermansia* and *Helicobacter* in EG2 compared with EG1 (Figure 3d).

### 3.4 Fecal concentrations of SCFA

**Figure 4:**
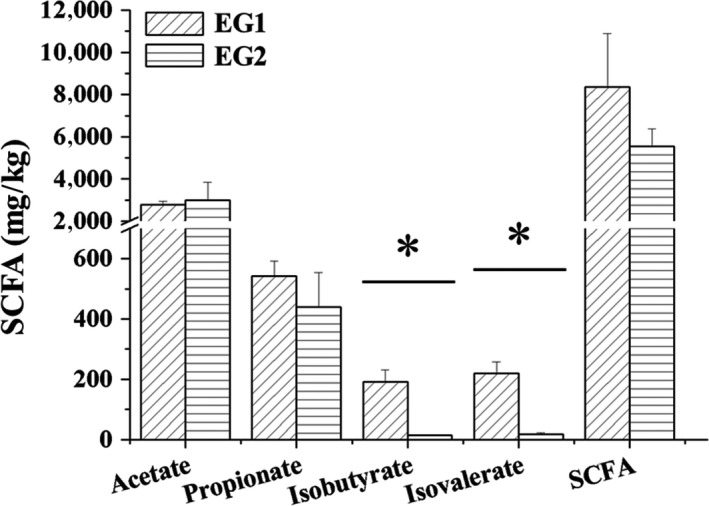
Mean concentrations of acetate, propionate, butyrate, isobutyrate, valerate, isovalerate, and total short chain fatty acids after lactulose intervention for 3 weeks. EG1, experimental group, week 0; EG2, experimental group, 3rd week, *n* = 6. Data were analyzed by nonparametric test followed by Mann–Whitney *U*‐test. * *p *<* *.05

SCFA concentrations in feces (mg/kg) were shown in Figure 4. After lactulose intervention (i.e., comparing EG2 with EG1), there was no significant variation in the concentrations of acetate, propionate, butyrate, and total SCFAs. However, levels of BCFAs were significantly decreased.

## 4 DISCUSSION

The dominant bacterial phyla detected in feces of mice were Bacteroidetes and Firmicutes, which were also previously detected in healthy mice (Chae et al., 2016; Mao et al., 2016). Bacteroidetes and Firmicutes are main groups of bacteria involved in metabolizing undigested food (Parkar, Trower, & Stevenson, 2013). The ratio of Firmicutes to Bacteroidetes significantly increased after lactulose intervention. An increased ratio of Firmicutes to Bacteroidetes was observed in patients with obesity (Ley, Turnbaugh, Klein, & Gordon, 2006). This ratio is also a useful process stability indicator in industrial applications, and has been used as a critical indicator in gut microbiome studies and gastrointestinal health evaluation (Chen, Cheng, Wyckoff, & He, 2016).

Lactulose intervention significantly increased the abundance of *Bifidobacteria* and Lactobacilli, which have a bifidogenic effect (Foster‐Fromme et al., 2011). This study was consistent with previous reports (Cho & Kim, 2014; Zhao, Li, Mohammadi, & Kim, 2016). The families Lactobacillaceae and Bifidobacteriaceae contain well‐known probiotic bacteria that benefit for human health. Those families are related to the production of energy in humans and animals by increasing the levels of SCFAs in the gut. SCFAs have been shown to regulate expression of genes by binding to G protein‐coupled receptors, and affect a wide range of biological functions (Puertollano, Kolida, & Yaqoob, 2014). SCFAs also result in a lower colon pH, which selectively stimulates growth of Bifidobacteria populations, inhibits the growth of potential pathogens, and modulates the immune system (Kaur & Gupta, 2002).

Lactulose intervention increased the abundance of some mucin‐degrading bacteria such as *Akkermansia* and *Helicobacter*, which were not able to metabolize lactulose but could use mucin as a carbon source (Mao et al., 2016). Some previous studies indicated that SCFAs were able to increase levels of mucin by lowering the pH (Barcelo et al., 2000). *Akkermansi*a, which is specialized for mucus degradation, is a genus in the phylum Verrucomicrobia (Tremaroli & Backhed, 2012). *Akkermansia* is important for our human health, and in the intestinal tract, may mediate obesity, diabetes, and inflammation (Derrien, Vaughan, Plugge, & De Vos, 2004); this genus also contributes to the restoration of antimicrobial peptides, for example, regenerating islet‐derived protein 3γ. *Helicobacter* was first cultivated from human gastric biopsy specimens in 1982 (Solnick & Schauer, 2001), and it has been linked to intestinal disease (Fox, 2002), but recent study indicated that colonization by *Helicobacter* species appeared to have no impact on the histopathology of liver or gut of possums (Coldham et al., 2013). Our previous study (Zhu et al., 2013) suggested that mucin‐degrading bacteria played an important role in maintaining the balance between mucin and SCFAs. Those data indicate that lactulose potentially improves gut health by stimulating mucin production to maintain the mucin‐SCFA balance.

**Figure 5:**
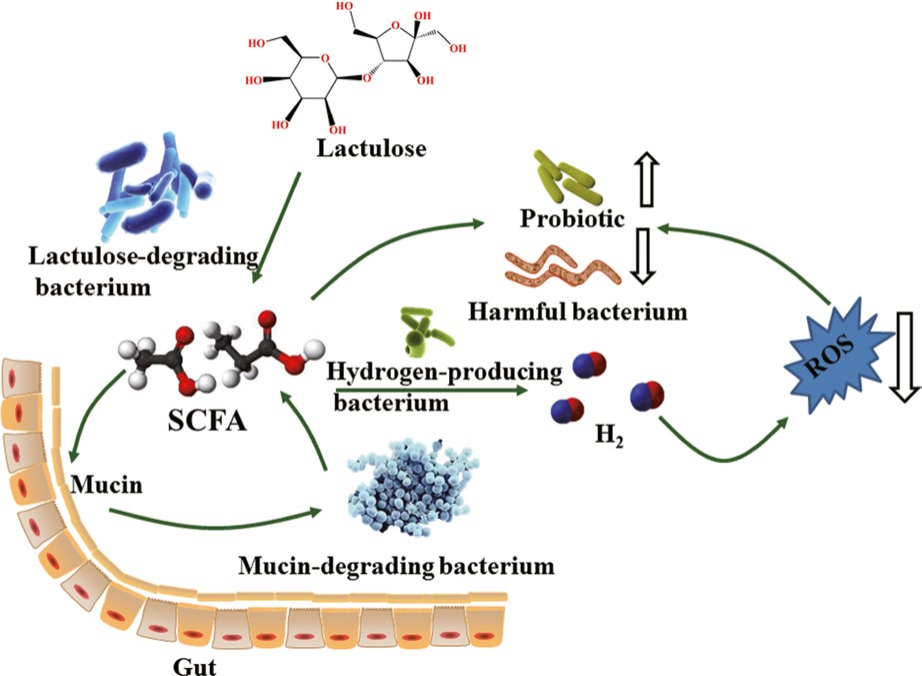
Effect of lactulose on gut microbiota. Lactulose intervention increased hydrogen‐producing bacteria, probiotics, mucin‐degrading bacteria, decreased pathogenic bacteria and harmful metabolites in mice

Lactulose intervention increased the abundance of some hydrogen‐producing bacteria such as Prevotellaceae and Rikenellaceae. Hydrogen can selectively neutralize cytotoxic reactive oxygen species and protect cells from oxidative stress injuries (Chen, Zuo, Hai, & Sun, 2011). Previous study suggested that lactulose reduced oxidative stress by producing hydrogen (Ghanizadeh, 2012). Chen et al. (2011) indicated that endogenous hydrogen could reduce oxidative stress and ameliorated symptoms of inflammatory bowel disease in humans. Lactulose increased the amount of intestinal hydrogen‐producing bacteria, thereby affecting intestinal oxidative stress. However, lactulose intervention significantly reduced the abundance of the family Desulfovibrionaceae, lipopolysaccharide‐producing bacteria, that are enriched in obese humans and rodents, and enhanced in all animals with impaired glucose tolerance (Zhang et al., 2010). The genus *Desulfovibrio* can decompose sulfur compounds in the gastrointestinal tract to hydrogen sulfide (H_2_S), which could damage the intestinal barrier, leading to a variety of diseases (Scott, Gratz, Sheridan, Flint, & Duncan, 2013). Endogenous H_2_S has a noxious effect on gut epithelial cells, hinders butyric acid oxidation, and causes apoptosis and chronic inflammation (Hulin et al., 2002). In this study, after lactulose intervention, the abundance of Desulfovibrionaceae decreased significantly; the reason may be that lactulose intervention decreased the colonic pH and changed the oxidation/reduction potential, making the intestinal tract unsuitable for Desulfovibrionaceae. Therefore, the action of lactulose as a prebiotic may be due to its ability to reduce the relative abundance of harmful gut microbiota (Figure 5).

In this study, the concentration of SCFAs in feces showed no significant change between EG1 and EG2, whereas BCFAs significantly decreased in EG2 compared with EG1. SCFAs have been shown to regulate inflammation, appetite and insulin resistance (Yang & Rose, 2016), which play a significant role in host health. Mucin‐degrading bacteria could produce SCFAs, whereas mucosa could absorb SCFAs, the balance between mucosa and SCFAs may explain why there was no significant difference in SCFAs concentrations between EG1 and EG2 (Zhu, Qin, Zhai, Gao, & Li, 2017). BCFAs are major markers of protein fermentation, which is likely to be detrimental to host health (Yang & Rose, 2015). Calik and Ergun (2015) found that there were no apparent differences in cecal acetate, propionate, butyrate, and total SCFAs, which was consistent with our result.

## 5 CONCLUSIONS

In this study, we evaluated the effect of lactulose on gut microbiota and SCFAs of C57BL/6J mice. Our findings suggested that lactulose could improve host health by selectively stimulating growth of the hydrogen‐producing bacteria Prevotellaceae and Rikenellaceae, probiotics Bifidobacteriaceae and Lactobacillaceae, and mucin‐degrading bacteria *Akkermansia* and *Helicobacter*, and decreasing the abundance of Desulfovibrionaceae and harmful metabolites. In addition, lactulose decreased the concentration of BCFAs, but maintained a stable concentration of total SCFAs. Our findings contribute important data on the interaction between lactulose and gut microbiota, and the mechanisms of why lactulose is beneficial for host health; nevertheless, further studies are needed to explain the datailed mechanisms and associated signaling pathways.

## CONFLICT OF INTEREST

The authors confirm no conflict of interest.
